# Circulatory MIC-1 as a Determinant of Prostate Cancer Racial Disparity

**DOI:** 10.3390/cancers12103033

**Published:** 2020-10-18

**Authors:** Dev Karan, Jo Wick, Seema Dubey, Ossama Tawfik, Peter Van Veldhuizen

**Affiliations:** 1 Department of Pathology, MCW Cancer Center and Prostate Cancer Center of Excellence, Medical College of Wisconsin, 8701 Watertown Plank Road, Milwaukee, WI 53226, USA; sdubey@mcw.edu; 2 Department of Biostatistics and Data Science, University of Kansas Medical Center, Kansas City, KS 66160, USA; jwick@kumc.edu; 3 Department of Pathology, Saint Luke’s Health System of Kansas City, Kansas City, MO 64111, USA; otawfik@saint-lukes.org; 4 Department of Internal Medicine, University of Rochester Medical Center, Rochester, NY 14642, USA; peter_vanveldhuizen@urmc.rochester.edu

**Keywords:** MIC-1, racial disparity, prostate cancer, biomarker

## Abstract

**Simple Summary:**

African American men are diagnosed with more aggressive prostate cancer and have worse outcomes than Caucasians. This study examined the role of MIC-1 as a risk factor and demonstrated a conceptual observation for the differential level of MIC-1 in circulation (serum and urine) and tumor tissues from prostate cancer patients of racial disparity. The circulatory MIC-1 levels in serum and urine are significantly higher in prostate cancer patients of African American ethnicity, with higher sensitivity and specificity than Caucasians. The validation of circulatory MIC-1 in a larger cohort of patients may help identify high-risk prostate cancer patients and develop race-oriented therapies to reduce the observed cancer outcome gaps between the races.

**Abstract:**

In this study, we investigated the potential of MIC-1 (macrophage inhibitory cytokine-1) on the severity of prostate cancer between African American men and Caucasians. Differences between the races were examined using Mann–Whitney tests for continuous variables and Fisher’s exact tests for categorical variables. Pearson’s correlation coefficient was used to identify associations between continuous measures across all samples and within each race. Analysis of variance, including clinical parameters, was used to identify differences in serum and urine MIC-1 levels between races. We found significant differences between the two races for age (*p* = 0.01), Gleason scores (*p* = 0.01), and stage of disease (*p* = 0.03). African American men in the study had higher Gleason scores (mean = 6.9) than Caucasians (mean = 6.5), during earlier stages of the disease. In Caucasian men with prostate cancer, serum MIC-1 expression was positively associated with age (*r* = 0.7, *p* < 0.01). However, African American men had highly expressed MIC-1 and high Gleason scores (*r* = 0.16, *p* = 0.3). Interestingly, the urine MIC-1 level was significantly higher in African American men with prostate cancer than in Caucasian patients. It appeared to be more sensitive and specific for African Americans (AUC = 0.85 vs. 0.56). Thus, high circulatory MIC-1 in prostate cancer patients may indicate MIC-1 as a potential biomarker to improve the diagnostic ability of an aggressive stage of prostate cancer in African American men. However, a larger cohort of sample analysis is required to validate these observations.

## 1. Introduction

Prostate cancer is the most common cancer and is the second leading cause of cancer-related deaths among men, accounting for almost 30,000 deaths annually in the United States [1]. Unfortunately, prostate cancer disproportionately affects African American men in both incidence and mortality rates compared to Caucasian men. Despite the emphasis on PSA-based screening and early diagnosis of prostate cancer, African American men are diagnosed with more aggressive disease and have worse clinical outcomes following treatment than Caucasians. At the time of diagnosis, African American men showed significantly worse pathologic outcomes after prostatectomy than their white counterparts, including higher risks of Gleason grade upgrading and positive surgical margins [2].

Although race is considered one of the contributing factors in prostate cancer, the molecular basis of racial disparities remains elusive. Several studies showed race-specific differential expression of CD24, SPINK1, PTEN deletion, ERG re-arrangements, and a higher telomere length associated with high-grade disease in prostate tumors [3,4,5,6]. Overexpression of miR-130b was also recognized as a contributor to prostate cancer racial disparity and was associated with poor prognosis in African American prostate cancer patients [7]. Khan et al. demonstrated that high levels of inhibitors of apoptosis proteins (IAPs) in the exosomal vesicles (EVs) from African American prostate cancer patients may influence tumor aggressiveness [8]. However, none of the molecular markers was found to be helpful in the clinical diagnosis of prostate cancer disparity among patients of a different ethnicity.

Macrophage inhibitory cytokine (MIC-1), as a growth differentiation factor (GDF15), is a member of the transforming growth factor (TGF)-β superfamily and is expressed in a variety of human tumor tissues. Multiple studies have highlighted the role of MIC-1 in association with the progression and metastasis of various cancer types. Previously, we also described the regulation of MIC-1 in association with inflammation and higher staining in prostate cancer tissues compared to benign prostate tissues [9]. Interestingly, increased MIC-1 expression is associated with the development and progression of prostate cancer, and rising serum MIC-1 levels are correlated with the presence of bone metastasis [10]. Further analysis supports the theory that serum MIC-1 improves the specificity for prostate cancer detection [11,12]. However, the role of MIC-1 in differentiating the nature of prostate cancer between African Americans and Caucasians remains mostly unknown.

In the present study, we analyzed the circulatory levels of MIC-1 in serum and urine from pre-operative prostate cancer patients. We also examined MIC-1 immunostaining in archival specimens of prostate tumors in both races. A high level of MIC-1 in samples from African American patients implicates the biological role of MIC-1 in the racial disparity of prostate cancer. The use of circulatory MIC-1 may improve the clinical diagnosis of aggressive prostate cancer, as is often seen in African American men.

## 2. Patients and Methods

### 2.1. Patients and Samples

A total of 145 serum and 39 urine samples from healthy donors and prostate cancer patients were tested. Serum samples for 80 prostate cancer patients (40 African American and 40 Caucasian men) collected during the time of diagnosis (pre-operative), and 40 archived samples from healthy donors (20 from each race), stored at −80 °C were acquired from the bio-specimens repository at the University of Kansas Cancer Center following approval from the Institutional Review Board and Ethics Committee (IRB00000161). Serum and urine samples for 15 Caucasian and 10 African American prostate cancer patients collected at the time of diagnosis and stored at −80 °C were obtained from Dr. Peter Van Veldhuizen (IRB00000161 and IRB00006196). Urine samples from 14 healthy African American male volunteers were collected in a de-identified manner, stored at −80 °C, and analyzed at the University of South Carolina School of Medicine as per approved protocol by the Institutional Review Board (Pro0004139).

### 2.2. Enzyme-Linked Immunosorbent Assay (ELISA)

Serum and urine MIC-1 level (pg/mL) was measured with sandwich ELISA using a commercially available ELISA assay kit (R & D Systems, MN). MIC-1 ELISA was performed twice in duplicates, and the four readings of MIC-1 for each patient were pooled to represent the amount of MIC-1 in the serum or urine samples from prostate cancer patients.

### 2.3. Immunohistochemistry (IHC) and Evaluation of Immunohistochemical Staining

Twenty-four prostate cancer samples, each from African American and Caucasian, were analyzed for MIC-1 protein expression using anti-MIC-1/GDF15 rabbit monoclonal antibody from Abcam. The archived specimens were obtained from the bio-specimen repository at the University of Kansas Cancer Center (Kansas City, KS, USA). Immunohistochemical analysis of MIC-1 protein expression was performed by a board-certified pathologist (OT) with specialized expertise in prostate cancer, as previously described [13]. Intensity and extent of MIC-1 staining were recorded with no prior knowledge of the patient’s history. To follow uniformity in scoring, each sample was given a composite score based on the intensity and extent of tissue staining by the pathologist. The intensity score was graded on a four-point scale corresponding to its numeric score, as in our previous study [13].

### 2.4. Statistical Analysis

Statistical analyses were performed using RStudio [14]. Natural log transformations were used to normalize the serum and urine values of MIC-1. We examined the differences between samples from African Americans and Caucasians using Mann–Whitney tests for continuous variables and Fisher’s exact tests for categorical variables. Pearson’s correlation coefficient was used to identify associations between continuous measures across all samples and within the race. Analysis of variance, including the factors age, race, and disease status, was used to identify adjusted differences in serum and urine MIC-1 levels between races. Receiver operating characteristic (ROC) curves were used to investigate the diagnostic ability of serum and urine MIC-1 expression for prostate cancer. All *p*-values reported are two-sided and are considered to be hypothesis-generating findings.

## 3. Results

### 3.1. Univariate Analyses

**Figure 1:**
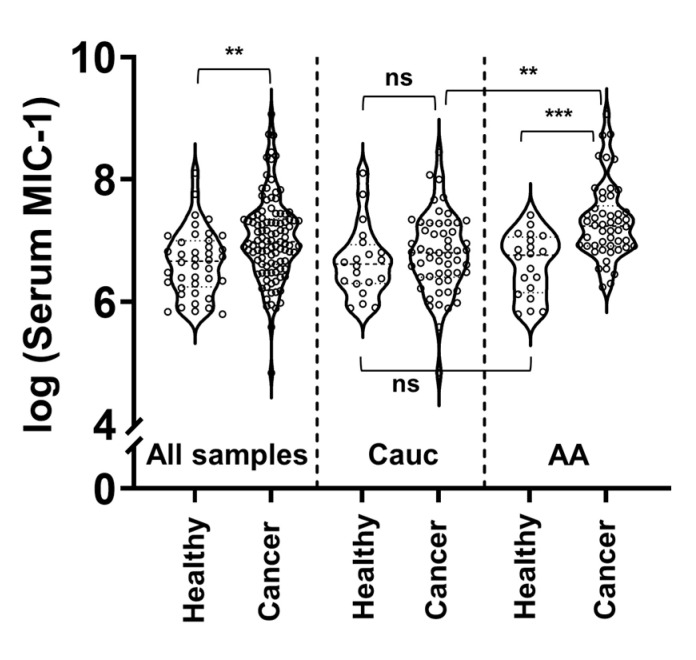
Violin plot for an overall comparison of serum MIC-1 levels (log transformed values) with individual data points between healthy and prostate cancer patients in Caucasians (Cauc) and African Americans (AA). All samples include both races (healthy: *n* = 40; Cancer: *n* = 105). Statistical significance assessed by using Tukey-adjusted *p*-values from ANOVA demonstrates *p* < 0.01 (**), *p* < 0.001 (***) for starred comparisons, and ns represents non significant.

The primary question addressed in this analysis is whether MIC-1 provides any predictive capability for prostate cancer severity at the time of initial diagnosis. Our data consists of information on 50 African American and 55 Caucasian men between the ages of 43 and 77 years (Median = 61 years). Because the African American race is a well-known risk factor for prostate cancer development, the severity of disease at diagnosis, and poor disease prognosis, we report the results both for the entire sample and by race (Table 1). The *p*-values in Table 1 were generated from the untransformed variables using nonparametric tests that do not assume normality of the data. Highly significant differences between the two races were found in age (*p* = 0.02), Gleason scores (*p* = 0.01), and stage of disease (*p* = 0.03). On average, African American men were 7 years younger than Caucasian men and had higher Gleason scores (mean = 6.9, SD = 0.7) than Caucasians (mean = 6.5, SD = 1.1), and earlier stage of the disease. PSA levels were also 23% higher (*p* = 0.09) in African American men (mean = 8.6, SD = 5.3) than in Caucasians (mean = 7.0, SD = 4.0). African American men, overall, had higher serum MIC-1 expression (median = 1152.2 pg/mL, range = 329.1 to 8682.5) compared to Caucasian men (median = 809.9 pg/mL, range = 126.3 to 4664.4). Serum MIC-1 levels in healthy subjects from both races were similar (*n* = 20 African American, median = 865.7 pg/mL, range = 329.1 to 1667.1; *n* = 20 Caucasian, median = 747.0 pg/mL, range = 364.5 to 3311.9; *p* = 0.8). However, serum MIC-1 from African American prostate cancer patients (median = 1,393.9 pg/mL, range = 512.2 to 8,682.5) was significantly higher as compared to African American healthy donors (median = 865.7 pg/mL, range = 329.1, 1667.1; *p* < 0.001) (Figure 1). This trend was not observed in Caucasian men (*p* = 0.3) (Figure 1).

### 3.2. Correlation Analyses

**Figure 2:**
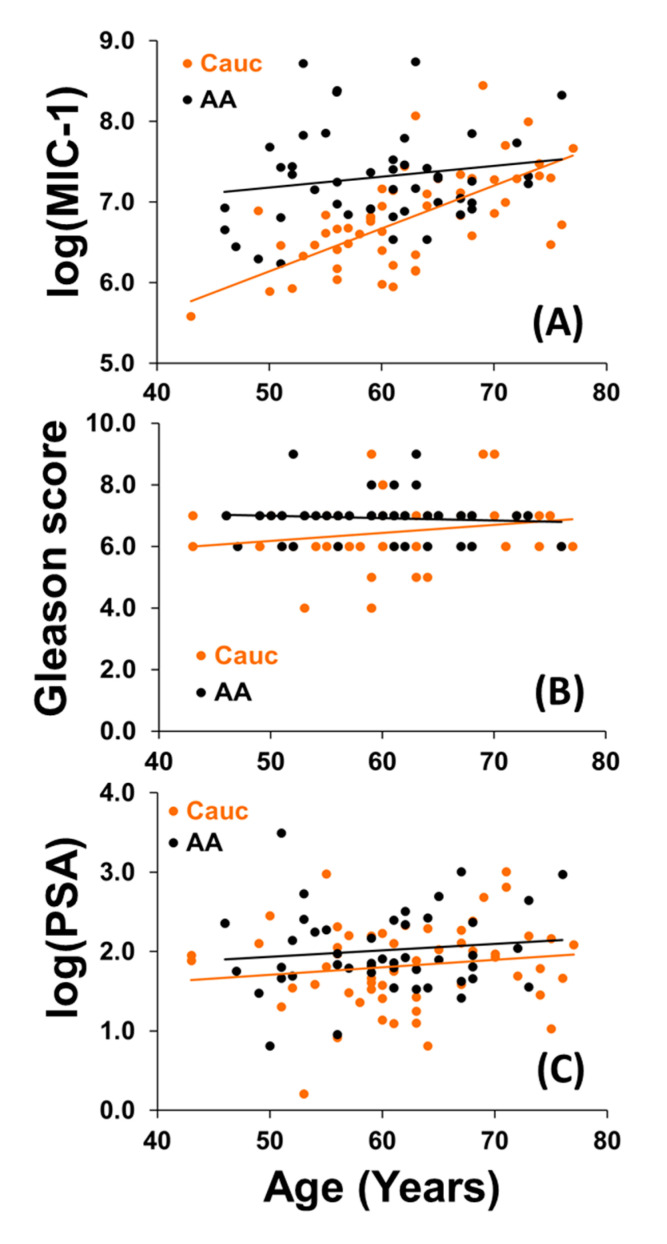
Scatterplots comparing correlative analysis for (**A**) log(MIC-1), (**B**) Gleason score, and (**C**) log(PSA) with age between the races.

Upon examination of the data, MIC-1 and PSA levels were found to be highly skewed. Due to the non-normality of these data, we performed natural log transformations on MIC-1 and PSA levels to meet the parametric assumptions required by correlation and regression analyses. Pairwise correlations were used to identify linear relationships between variables. Pearson’s correlation coefficient is provided in Table 2 for age, log(PSA), and log(MIC-1). Because the Gleason score is ordinal over the range 6 to 9, Spearman’s nonparametric correlation coefficient was used to calculate bivariate correlations that included the measure. Among African Americans, no significant correlations were found among any of the variables. However, in Caucasians log(MIC-1) serum was found to be positively associated with log(PSA) (*r* = 0.26) and age (*r* = 0.67), while the Gleason score was positively associated with log(PSA) (*r* = 0.21) and age (*r* = 0.20). Thus, higher levels of MIC-1 and higher Gleason scores are associated with older patients when limiting our sample to Caucasians. However, this was not true in African Americans, as both older and younger patients have highly expressed MIC-1 (*r* = 0.35) (Figure 2A) and high Gleason scores (*r* = −0.08) (Figure 2B). This phenomenon was not observed when examining PSA expression levels by age among African Americans and Caucasians. Though it appears that African Americans have slightly higher PSA levels than Caucasians when accounting for age, there is no age association of PSA in either of the groups (Figure 2C).

### 3.3. Multivariable Regression Models

To further examine these differences at the levels of the categorical variable such as race, we used analysis of covariance (ANCOVA), which combines regression and analysis of variance by introducing the continuous variable, age, into the ANOVA model. Because we noticed that the slopes of the regression lines within the two groups were not parallel for serum MIC-1 (Figure 2A), we used a model that included an interaction term with the race to determine whether the slopes of the lines differ significantly by race. The slope of the regression line for Caucasians was significantly more positive in the log(MIC-1) when compared to that of African Americans (*p* < 0.01, Figure 2A); that is, the association of age with MIC-1 is significantly greater in Caucasians than in African Americans. Additionally, from a centered-age model, we found that Caucasians at 61 years of age have significantly reduced log(MIC-1) expression levels (mean = 6.72, 95% CI = 6.58, 6.87) when compared to African Americans of the same age (mean = 7.33, 95% CI = 7.17, 7.48) (*p* < 0.001). We built a similar model to adjust the comparison of log serum MIC-1 between races for Gleason score. The centered-Gleason Score model indicated that Caucasians with a Gleason Score of 6.7 have significantly reduced log(MIC-1) expression levels (mean = 6.81, 95% CI = 6.63, 6.98) when compared to African Americans with the same Gleason Score (mean = 7.29, 95% CI = 7.10, 7.48).

To further understand the association of serum MIC-1 level with Gleason scores (GS), we categorized these samples into two groups of GS ≤ 6 and GS ≥7 and performed ANOVA. Based on Tucky-adjusted p-values for multiple comparisons for all samples, the serum MIC-1 level was significantly higher (*p* = 0.02) in patients grouped in GS ≥ 7 (*n* = 67; mean = 1669.57 pg/mL) compared to GS ≤ 6 (*n* = 38; mean = 1079.71 pg/mL). Within the races, differences in the serum MIC-1 level between two GS groups were not statistically significant (AA: *p* = 0.41; Cau: *p* = 0.23). However, the comparison between the races for GS ≥7 showed significantly higher serum MIC-1 (*p* = 0.031) in African American prostate cancer patients than Caucasians.

A similar ANCOVA model for Gleason score, including the factors of age and race, showed no differences in the relationships between age and Gleason score for Caucasians and African Americans (*p* = 0.14, Figure 2B). Gleason scores differed significantly by race (*p* = 0.01), however, with Caucasians having lower Gleason scores (mean = 6.46, 95% CI = 6.23, 6.70) when compared to African American men of a similar age (mean = 6.91, 95% CI = 6.66, 7.17). One possible explanation for the lack of age-Gleason score association among African Americans is the lack of variability in Gleason scores for the African Americans in our samples. The range of scores varied only from 6 to 9, with greater than 80% of subjects having a 7 or higher score. An ANCOVA model utilizing a parallel-slope assumption was also used for log(PSA) based on the parallel lines found in Figure 2C. From this model, a significant racial difference was found in PSA levels (*p* = 0.04), with African Americans having higher PSA levels (mean = 7.54, 95% CI = 6.49, 8.85) than Caucasians across all ages (mean = 5.87, 95% CI = 5.05, 6.89). Additionally, no effect of age was found, indicating that there is no significant association between age and PSA levels, as was previously demonstrated (*p* = 0.18).

### 3.4. Analysis of urine MIC-1

**Figure 3:**
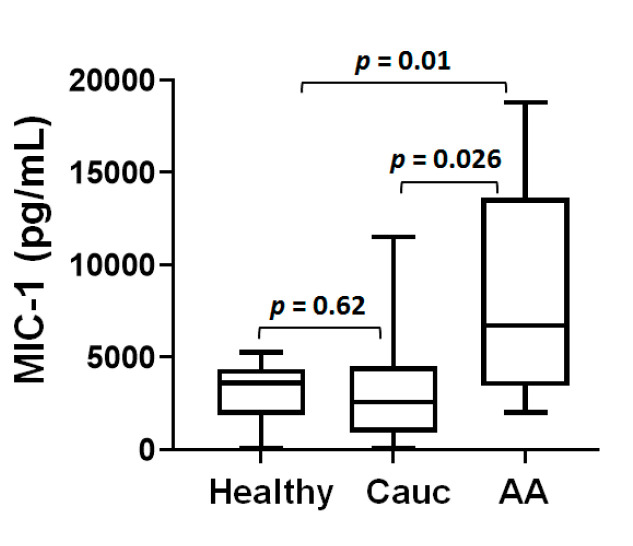
Box and Whisker plot for urine MIC-1 from African American healthy donors and prostate cancer patients. Samples include healthy (urine from African American men, *n* = 14), and prostate cancer patients from Caucasian (*n* = 15) and African American (*n* = 10) ethnicity. Statistical significance assessed by Tukey-adjusted *p*-values from ANOVA.

To further examine the role of MIC-1 for its predictive utility in the disparity of prostate cancer, we had access to the limited number of urine samples collected at the time of diagnosis from prostate cancer patients (African American = 10; and Caucasians = 15), whereas healthy donors (African American; *n* = 14) served as the control (Figure 3). A comparative analysis among urine samples showed significantly higher urine MIC-1 level in prostate cancer patients of African American race (median = 5959.4, range = 1564.0 to 19,411.8) as compared to Caucasian patients (median = 3263.6, range = 356.8 to 10,481.8; *p* = 0.03) or healthy African American donors (median = 3601.8, range = 94.9 to 5273.7; *p* = 0.01). Similar to serum analysis, we used pairwise correlations to examine linear relationships between clinical variables (age, PSA, Gleason score, and ln(MIC-1)). In both races, no significant association of urine ln(MIC-1) between clinical variables was observed (Table 2). This lack of correlation could be due to the limited number of urine samples.

### 3.5. ROC Curve Analysis of Circulatory MIC-1

**Figure 4:**
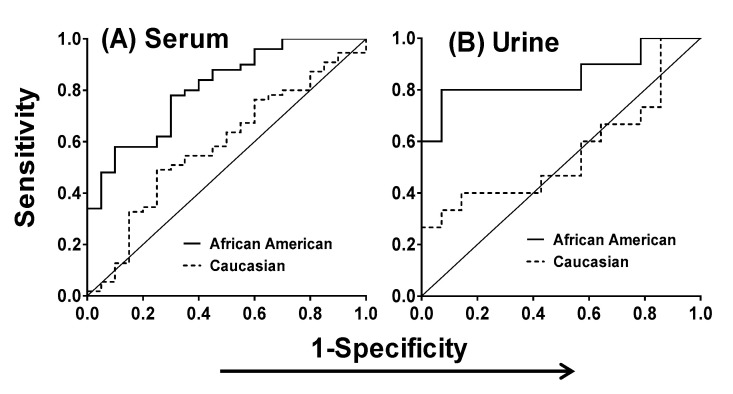
Receiver operating curve (AUC-ROC) analysis for MIC-1 in serum (**A**) and urine (**B**) samples from prostate cancer patients and healthy controls between the races.

To examine the potential of MIC-1 as a diagnostic tool for prostate cancer between the races, we compared the serum and urine MIC-1 observations using the area under the receiver operating curve (AUC-ROC). The cutoff value was chosen based on the highest Youden’s Index calculated based on the predicted values of sensitivity and specificity from the ROC analysis. Using a cutoff value of 904.6 pg/mL, the overall sensitivity and specificity of serum MIC-1 was 67.6% and 65%, respectively for serum MIC-1. For Caucasians, the predicted MIC-1 cutoff was 819.5 pg/mL with 54.5% sensitivity (95% CI = 40.5%, 68.0%) and 65% specificity (95% CI = 40.8%, 84.6%). In African Americans, the predicted MIC-1 cutoff was 995.2 pg/mL with 78.0% sensitivity (95% CI = 64.0%, 88.5%) and 70% specificity (95% CI = 45.7%, 88.1%) (Figure 4A).

For urine samples, a cutoff value of 4518 pg/mL yielded an overall sensitivity and specificity of 56% and 85.7%, respectively. In Caucasians, the MIC-1 cutoff was 5088 pg/mL with sensitivity and specificity of 26.7% (95% CI = 7.8%, 55.1%) and 92.9% (95% CI = 66.1%, 99.8%), respectively. In African Americans, the cutoff value of MIC-1 was 4969 pg/mL which provided a sensitivity of 80% (95% CI = 44.4%, 97.5%) and a specificity of 92.9% (95% CI = 66.1%, 99.8%) (Figure 4B). A comparison of AUC-ROC analysis between the races showed higher sensitivity and specificity of MIC-1 as a potential diagnostic tool for African American patients than for Caucasian prostate cancer patients.

### 3.6. MIC-1 Protein Expression in Prostate Tumor Biopsies

**Figure 5:**
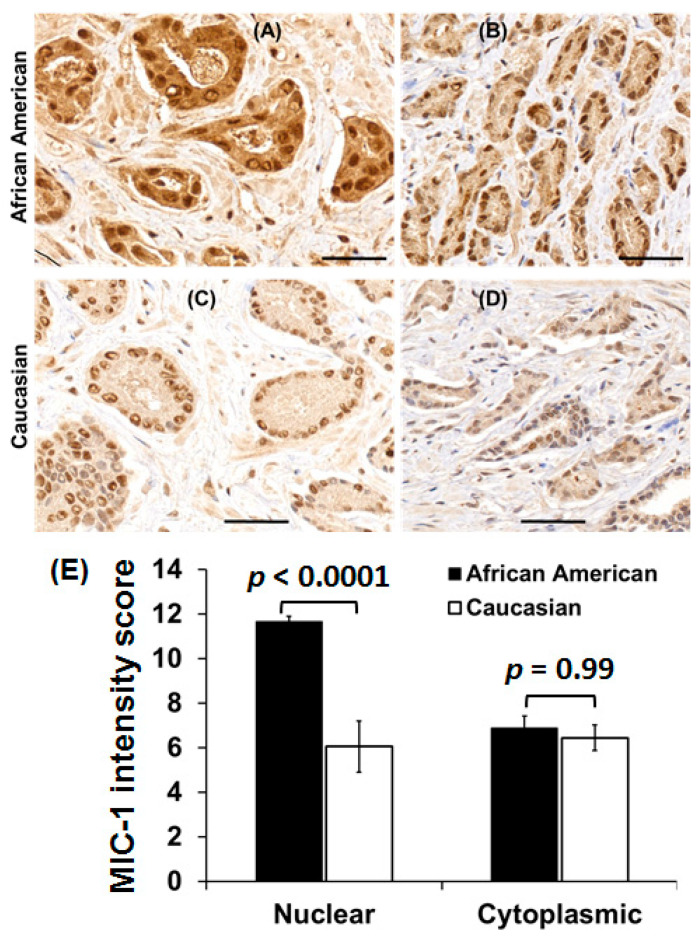
MIC-1 expression in prostate tumor tissues. Representations of high (**A**,**B**) and low (**C**,**D**) nuclear MIC-1 expression in prostate tumor tissues from African American men and Caucasians (*n* = 18); and (**E**) overall MIC-1 intensity score. The values represent standard error from the mean (mean ± SE). Statistical significance assessed by using Tukey-adjusted *p*-values from ANOVA. Scale bars represent 50 µm.

We further examined the expression of MIC-1 protein in archival prostate tumor specimens from both races (*n* = 24) using IHC. Out of 24 Caucasian prostate tumor specimens, six lacked cancer regions, while in African American specimens, three samples did not have cancer regions. Therefore, we scored MIC-1 immunostaining intensity in 18 specimens for both races (Figure 5). Interestingly, we observed a significantly higher (*p* = 0.0001) MIC-1 nuclear staining in African American specimens (intensity score 11.67 ± 0.24) as compared to Caucasians (intensity score 6.06 ± 1.15), while MIC-1 cytoplasmic level was similar in both races (6.89 ± 0.54 vs. 6.44 ± 0.57; *p* = 0.429). In African American specimens, 85–100% of cells showed MIC-1 staining. In contrast, in Caucasians, the percentage of nuclear staining cells was highly variable (ranged from 2% to 100%), and almost 100% of the cells showed weak to moderate MIC-1 cytoplasmic staining. In Caucasian specimens, 14/24 lacked benign tissue; therefore, comparison among races for the MIC-1 protein in adjacent benign tissue was omitted.

## 4. Discussion

Without considering race, several studies have proposed serum MIC-1 as a prognostic and predictive biomarker. Based on a prospective cohort study of Swedish men with a confirmed diagnosis of prostate cancer, elevated serum MIC-1 predicted poor cancer-specific survival [15]. A p-Chip-based immunoassay on 70 serum samples showed that MIC-1, combined with PSA, improved the specificity of prostate cancer diagnosis [16]. The current study showed a differential level of MIC-1 both in circulation and in tumor tissues from prostate cancer patients of African American and Caucasian races and proposes circulatory MIC-1 as a biomarker to improve clinical sensitivity in the diagnosis of prostate cancer in African American men.

MIC-1 level both in tissues and in plasma is usually low under normal conditions. However, it increases during inflammation or malignancy, including in the pathogenesis of cancer [9,17,18]. A tissue microarray-based immunostaining of prostate tumors showed a stage-wise decrease in MIC-1/GDF15 expression in African American men, but there was no change in MIC-1/GDF15 expression by higher grade. The overall MIC-1/GDF15 was elevated in prostate cancer compared to benign controls [19]. Interestingly, an increase in NF-κB was observed in association with increasing grade of prostate tumors in African American men. Although this study did not differentiate the nuclear and cytoplasmic staining of MIC-1/GDF15 and NF-κB, we observed a significantly higher nuclear MIC-1 protein in prostate tumors of African American men compared to Caucasians, while the cytoplasmic MIC-1 protein remained similar in both races. Increased MIC-1 level has been linked with NF-κB activation in the prostate cancer cell lines model [20]. During the transition of disease from prostatic intraepithelial neoplasia to prostate cancer, translocation of NF-κB to the nucleus was associated with biochemical recurrence of prostate cancer [21]. Pancreatic adenocarcinoma study also revealed that the activation of NF-κB directly regulates the MIC-1/GDF15 expression in tumor development [22]. Therefore, the observed high level of nuclear MIC-1 in African American prostate tumors may drive aggressive prostate cancer in association with NF-κB activation implicating a biological role of MIC-1 in the racial disparity of prostate cancer.

This study is the first exploratory report demonstrating a differential level MIC-1 in circulation (serum and urine) and tumor tissues from prostate cancer patients of racial disparity. The circulatory MIC-1 levels in serum and urine are significantly higher in prostate cancer patients of African American ethnicity, with a higher AUC-ROC than in Caucasians. These observations suggest that the combined utility of circulated MIC-1 may add to the diagnostic assessment of aggressive prostate cancer, as is often reported in African American men. The association of the PSA and Gleason scores with the clinical state of the disease has been well characterized. However, statistical analysis of age with the PSA and Gleason scores is often neglected in prostate cancer studies. A study involving 110 Brazilian Indian triable men of Macuxi ethnicity found a strong association of serum PSA (free-PSA and total-PSA) with the age groups of 60-79 year old patients [23].

Similarly, multiple studies analyzing different disease conditions, including cancer, showed MIC-1 association with age [24,25,26]. Previously, it was reported that the MIC-1 level increases in response to injury or inflammation and that the age-associated inflammation may lead to high MIC-1 in the circulation [17,27]. We also found an elevated MIC-1 level correlated with age and PSA in Caucasian prostate cancer patients. However, no correlation was observed when restricted to African American samples, suggesting MIC-1 as an independent predictor of prostate cancer in African American men. This study is limited to a small number of patient samples, specifically, urine-based observations. If validated in a large cohort of samples, circulatory MIC-1 may help to identify high-risk prostate cancer patients and to develop treatment strategies to reduce the observed cancer outcome gaps between the races.

## 5. Conclusions

Our observations suggest that serum and urine MIC-1 are significantly higher in African Americans with prostate cancer than Caucasians. Among African Americans, Gleason scores and MIC-1 levels were found to be independent of age. A comparison of serum MIC-1 level in patients within the category of GS ≥ 7 between the races showed a significantly high MIC-1 (*p* = 0.031) in African American patients. While an increase in nuclear MIC-1 protein in prostate tumors of African American patients suggests its biological role, high AUC-ROC (serum and urine), sensitivity, and specificity indicate the use of circulatory MIC-1 as a potential biomarker to improve the clinical diagnosis of prostate cancer in African American men. Another critical observation to be examined further is whether accumulated nuclear MIC-1 helps predict the aggressive prostate cancer for its subsequent biochemical recurrence. However, a larger cohort of study is needed to establish a cutoff range of MIC-1 as a diagnostic tool to improve the clinical sensitivity of prostate cancer.

## Figures and Tables

**Table 1:** Clinical characteristics and the disease status in prostate cancer patients and the control subjects (healthy donors) used for the serum and urine MIC-1 analysis.

Clinical Characteristics		All Samples (*n* = 159)	African American		Caucasian		*p* ^c^
			Case (*n* = 50)	Control (*n* = 34)	Case (*n* = 55)	Control (*n* = 20)	
PSA (ng/mL) ^a^		7.8 (4.7)	8.6 (5.3)	—	7.0 (4.0)	—	0.09
Age (years) ^a^		55.6 (14.0)	59.9 (7.4)	40.7 (19.0)	62.0 (8.1)	52.6 (10.7)	0.02
Gleason Score ^a^		6.7 (0.9)	6.9 (0.7)	—	6.5 (1.1)	—	0.01
Stage ^b^	T1	2 (2.1)	2 (4.0)	—	0 (0)	—	0.03
	T1a	1 (1.1)	1 (2.0)	—	0 (0)	—	
	T1c	6 (6.4)	6 (12.0)	—	0 (0)	—	
	T2	1 (1.1)	1 (2.0)		0 (0)		
	pT2a	12 (12.8)	4 (8.0)	—	8 (18.2)	—	
	pT2b	3 (3.2)	2 (4.0)	—	1 (2.3)	—	
	pT2c	62 (66.0)	33 (66.0)	—	29 (65.9)	—	
	pT3a	4 (4.3)	1 (2.0)	—	3 (6.8)	—	
	pT3c	2 (2.1)	0 (0)	—	2 (4.5)	—	
	pT4a	1 (1.1)	0 (0)	—	1 (2.3)	—	
Missing		11 (10.5)	0 (0)		11 (20.0)		

^a^ Mean (SD); ^b^ N (% of non-missing); and ^c^ *p*-values represent a comparison of African American and Caucasian samples via Mann–Whitney or Fisher’s exact test. In total, there were 184 observations for MIC-1 analysis from both races (145 for serum and 39 for urine). However, 25 samples were matched (common for serum and urine); therefore, the entire samples analysis with clinical characteristics includes 159 samples.

**Table 2:** Correlations (*r*) of clinical parameters with log(MIC-1) serum and urine expression in prostate cancer patients.

Parameters	All Samples		African American		Caucasian	
	logSerum MIC-1 (units)	logUrine MIC-1 (units)	logSerum MIC-1 (units)	logUrine MIC-1 (units)	logSerum MIC-1 (units)	logUrine MIC-1 (units)
logSerum MIC-1 (units)	—	—	—	—	—	—
logUrine MIC-1 (units)	*r* = 0.48 *p* = 0.02 *n* = 25	—	*r* = 0.46 *p* = 0.19 *n* = 10	—	*r* = 0.35 *p* = 0.2 *n* = 15	—
Age (years)	*r* = 0.35 *p* < 0.01 *n* = 105	*r* = 0.12 *p* = 0.57 *n* = 25	*r* = 0.16 *p* = 0.27 *n* = 50	*r* = -0.12 *p* = 0.74 *n* = 10	*r* = 0.67 *p* < 0.01 *n* = 55	*r* = 0.41 *p* = 0.13 *n* = 15
PSA (units)	*r* = 0.19 *p* = 0.05 *n* = 105	*r* = 0.16 *p* = 0.45 *n* = 25	*r* = 0.04 *p* = 0.78 *n* = 50	*r* = 0.37 *p* = 0.29 *n* = 10	*r* = 0.26 *p* = 0.06 *n* = 55	*r* = 0.01 *p* = 0.99 *n* = 15
Gleason Score	*r =* 0.25 *p* = 0.01 *n* = 105	*r =* 0.15 *p* = 0.46 *n* = 25	*r* = 0.11 *p* = 0.47 *n* = 50	*r* = 0.36 *p* = 0.31 *n* = 10	*r* = 0.22 *p* = 0.11 *n* = 55	*r* = −0.15 *p* = 0.59 *n* = 15
